# Non-obstetric vulva haematomas in a low resource setting: two case reports

**DOI:** 10.11604/pamj.2019.33.314.19488

**Published:** 2019-08-20

**Authors:** Ako Annabel Mangwi, Peter Vanes Ebasone, Desmond Aroke, Larry Tangie Ngek, Ako Simon Nji

**Affiliations:** 1 Anako Mother and Child Medical Centre, Yaoundé, Cameroon; 2 Clinical Research Education Networking and Consultancy, Yaoundé, Cameroon; 3 Health and Human Development Research Network, Douala, Cameroon; 4 Faculty of Medicine and Biomedical Sciences, Yaoundé, Cameroon

**Keywords:** Non-obstetric haematomas, vulva haematoma, Cameroon, conservative management, surgical management

## Abstract

Vulva haematomas are uncommon outside the obstetric population, with an incidence of 3.7% and represent only 0.8% of all gynaecological emergencies. The first case is a 24-year-old G2P1011 referred after the failure of conservative management of a progressively increasing right labia majora swelling. Vulva incision, exploration and relieve of hematoma were done under local anaesthesia. The second case is a 17-year-old G1P1001, a student who presented with spontaneous pain and swelling of the left labia majora. The swelling was rapidly increasing, tense and tender. It spontaneously ruptured, clots were drained and the wound was packed. Vulva hematomas are not very common hence necessitating careful assessment, right diagnosis and management. Management could be conservative (analgesics, local compression) as well as surgical in cases of hemodynamic instability, rapidly increasing size of hematoma and pain intensity. Prompt surgical management reduces the risk of infection and longer hospital stays, which is important in low resource settings like ours.

## Introduction

Vulva hematomas are uncommon outside the obstetric population and makeup 0.8% of all gynecologic admissions [1]. In the obstetric population, the incidence is 1-2 per 1000 deliveries, following episiotomy repairs and birth-related soft tissue injury [1,2]. In the non-obstetric population it may be due to trauma to the pelvis and or perineum sustained during a fall from a height, a saddle injury, sexual assault, insertion of a foreign body and coitus [2-4]. Vulva hematomas can also be as a result of a spontaneous rupture of blood vessels in the perineum. This can be as a result of aneurysms and varicosities. Blood vessels in the perineum are prone to damage from injuries of the vulva tissues against the pelvic bones. Although vulva hematomas can rapidly grow in size causing hemodynamic instability or can become infected, most are small and rarely pose a threat to the patient [3]. Bleeding could be venous in origin, but when it is arterial, it originates from one of the branches of the pudendal artery, and not the artery itself. This could be the transverse perineal, posterior labial or posterior rectal branches. Non-obstetric vulva hematomas are uncommon and even when they do occur, they are rarely reported in resource constraint settings. We herein present 2 cases of non-obstetric vulva hematomas.

## Patient and observation

**Figure 1:**
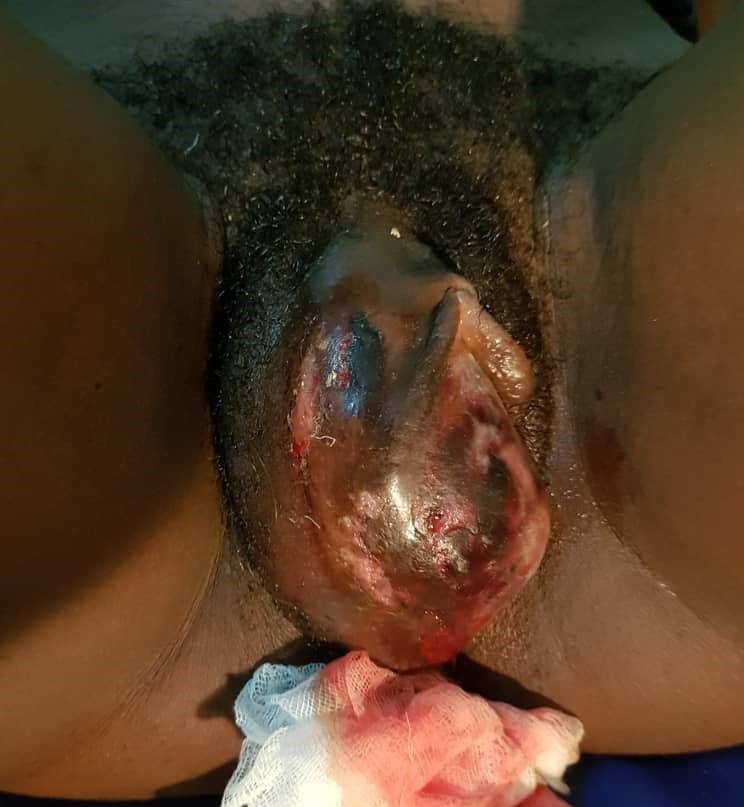
Right necrotic vulva hematoma

**Figure 2:**
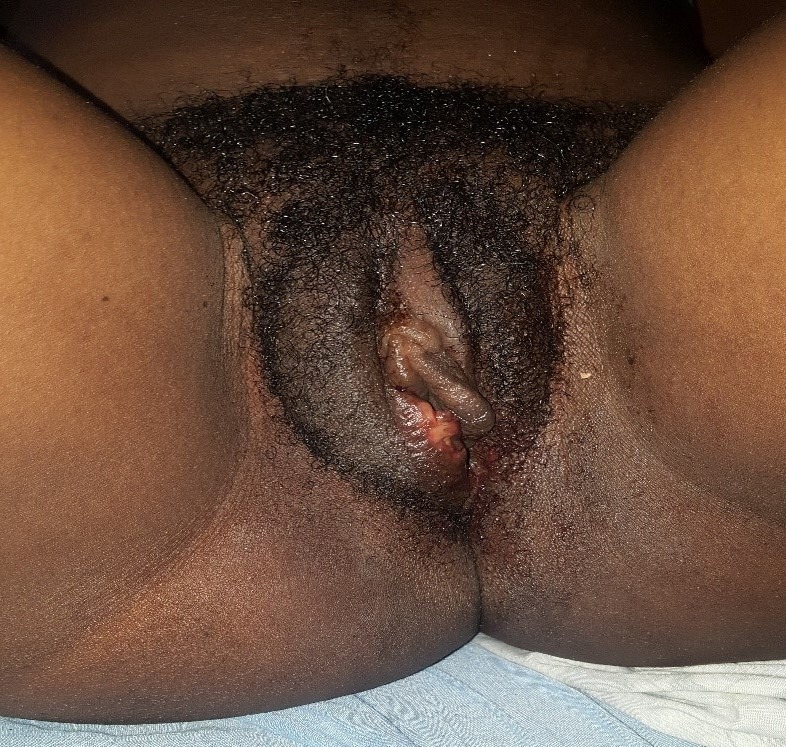
After resolution of Hematoma

**Case 1**: a 24-year-old female was referred to ANAKO Mother and Child Medical Centre on the 07/12/2019 from a 280km locality for better management of a large swelling on the right labia. Three days prior to arrival (05/12/2018), she noticed gradual onset, painful and progressive swelling on the right side of her vulva. The swelling started 5 hours following coitus with her usual partner. She denied aggression, coercion, instrumentation with other objects or use of drugs by her partner during or prior to coitus. She had no history of easy bruising or excessive bleeding after trauma. The patient went to a nearby health facility where she was hospitalized. She was conservatively managed with antibiotics and transfused 450cc of whole blood. Progress was marked by intermittent bleeding and an increase in the size of the swelling. An increase in the intensity of the pain prompted a referral to our health facility. She was G2P1011. She terminated a pregnancy at 8 weeks, 3 years earlier. A live vaginal birth 15months earlier with neither vaginal nor perineal tears. Menarche was at 14 years; she bleeds for 5 days and has a regular cycle of 30 days. On examination, the patient was alert. Her blood pressure was 126/78 mmHg, heart rate was 90 beats per minute, respiratory rate was 18 breaths per minute and her temperature was 36.5°C. She had mild pallor. On palpation of her abdomen, no tenderness, guarding, or rebound was elicited. At the perineum, there was a large swelling on the right labia majora, measuring 7x6 cm, necrotic, tender and tense, a vaginal examination was not possible (Figure 1). A pelvic (transabdominal) ultrasound was done which showed pelvic organs in place and a hematoma thus eliminating a uterine prolapse. A full blood count was done which revealed a normocytic normochromic anaemia at 9g/dl. Platelets were normal. Vaginal examination/exploration and debridement were done under local anaesthesia and a packed dressing was done (Figure 2). No further bleeding was noted after this. She was given the following medications, antibiotics; ceftriaxone 1g every 12 hours, metronidazole 500mg every 08 hours and gentamycin 80mg every 12 hours, analgesics: paracetamol 1000mg, 08 hourly. She was also supplemented with iron tablets, 80mg/day. Sitz baths were started and done 12 hourly and polyvidone iodine. She was discharged on day three post admission and continued with polyvidone iodine ovules and sitz baths. Subsequent follow up was unremarkable.

**Figure 3:**
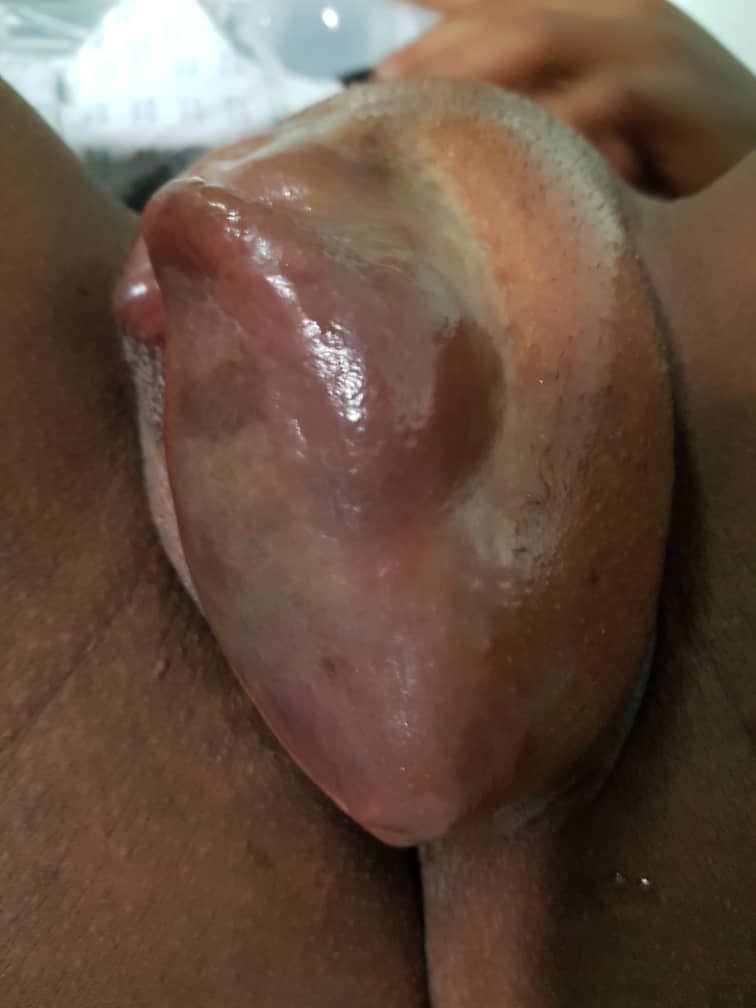
Right vulvar haematoma

**Figure 4:**
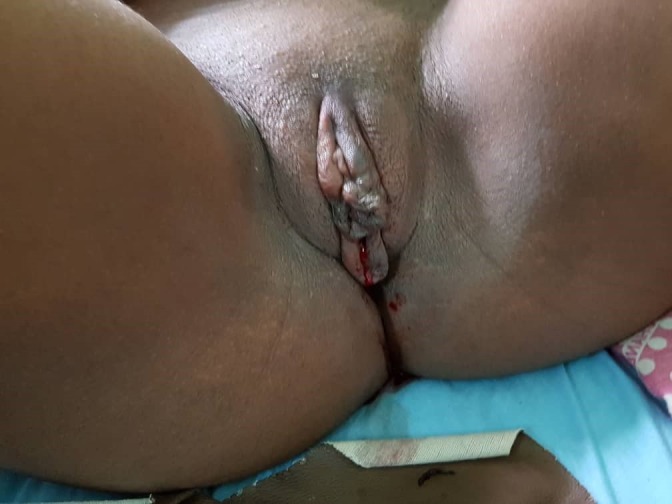
After resolution of hematoma

**Case 2:** a 17-year-old female presented to our emergency room on the 01/12/2018 with pain and swelling on the left labia majora. It started 3 hours before arrival. She noticed a rapidly increasing swelling which was extremely tender and tense on the left side of the vulva. She was unable to stand or sit and the pain was not relieved by painkillers, paracetamol and diclofenac. There was no history of coitus in the last 3 days and she had shaved the previous day. She had no history of easy bruising or excessive bleeding after trauma. She is G1P1 and mother to 7-month-old infant. On arrival, she was in pains. Her blood pressure was 130/87 mmHg, a pulse of 78 beats per minute and temperature was 37.2°C. Conjunctivae were pink and sclera anicteric. There was a large swelling on left labia majora 9x7 cm, tense, tender with no skin discoloration (Figure 3). The swelling ruptured spontaneously and we drained about 500cc of clotted blood. The wound was packed and there was no further bleeding until 12 hours later when the dressing was removed (Figure 4). She received prophylactic antibiotics; ceftriaxone 2g before debridement and had fluids, normal saline and ringers lactate, 500cc 12 hourly for 24 hours. The patient was discharged two days later with polyvidone iodine ovules and sitz baths.

## Discussion

The vulva is the external part of the female genitalia. It protects the urinary opening, vestibule and the vagina. It is largely made up of smooth muscle and loose connective tissue. Its arterial supply is from the internal iliac artery through several branches of the pudendal artery [4]. The vulva is drained by branches of the internal pudendal vein, venae comitantes. This rich blood supply makes the vulva vulnerable to hematoma formation following a blunt injury to the perineum. In strictly vulva hematomas, the bleeding is restricted above the anterior urogenital diaphragm, while in vulvovaginal haematomas the bleeding extends to the paravaginal tissues [5]. A good history and physical examination are important to rule out differential diagnoses such as Bartholin’s gland abscesses and cysts, vulva varicosities and folliculitis. Vulva haematomas of non-obstetric origin are relatively rare, with an incidence of 3.7% and represent only 0.8% of all gynecological emergencies [6,7]. Some common causes include straddle type injuries, coitus or physical assault [8]. In the absence of evident trauma, spontaneous vessel rupture is considered as the cause [1]. In our first case, vulva hematoma was a result of a coital injury. It is reported that the most common causes of non-obstetric injuries to the genital tract are due to coitus, making up about 40% of such admissions [7]. Although there is no anatomical explanation, 70% of all reported vulva hematomas appear on the right labium as in our first case [9]. Because cases are relatively rare, there is no clear-cut consensus on the management of vulva haematomas. Current management modalities include; conservative management, surgical management and selective arterial embolization. The choice of management depends on the size of the haematoma, the involvement of adjoining organs and the degree of haemodynamic stability. This can be adequately visualized with transperineal ultrasound, a very useful tool when doing expectant management [10].

Conservative management is advocated for small haematomas with no acute expansion [8,11,12]. Patients who are managed conservatively are more likely to spend more days in admission, need more antibiotics and blood transfusion [13]. This is typical of our first case, prior to arriving at our service she was managed with antibiotics, blood transfusion and ended up spending more days in more than one hospital. Conservative management is usually done with ice packs, bed rest, analgesics and local compression. As pressure builds within the haematoma, the tissue becomes necrotic, necessitating debridement and prevention of infection [1]. Surgical intervention is recommended if haematoma keeps increasing and the pain persists, usually marking the failure of the conservative method [6,11]. In our second case, the haematoma was rapidly increasing and eventually ruptured, it was absolutely necessary for us to carefully remove all clots and assess for pressure necrosis. Surgery involves the evacuation of blood clots and ligation of bleeding vessels. Since the most affected vessels are veins, ligation is often less likely. In both cases, we evacuated blood clots, exploring the haematoma to evaluate its extent but careful enough not to cause further bleeding. Selective arterial embolization is becoming a new way of managing these lesions. This procedure was first described in 1979 by Brown *et al.* [1]. It is advantageous because it results in shorter hospitalization days compared to surgical management [11]. Arterial embolization is a costly technical procedure, requiring expertise and skills that are scarce in our setting. In both of our cases, it was not an option because of the unavailability of the procedure and the financial status of the patients. More so, our patients had already existing hematomas that required evacuation.

## Conclusion

Non-obstetric vulva hematomas are uncommon and potentially life-threatening conditions necessitating a careful assessment and timely surgical intervention. Most cases can be managed conservatively, but attention must be paid to haematoma size, signs of haemodynamic instability, increasing pain and necrosis as this often necessitates an urgent surgical intervention. Prompt surgical intervention reduces risk of infection, longer hospital stays and death. Since the condition is relatively rare, doctors need to be alert to identify when conservative management is insufficient or inappropriate, considering surgical intervention timely. This is particularly important in low resource settings where patients often have to travel long distances to access surgical management.

## Competing interests

The authors declare no competing interests.
